# Quantifying Influences on Intragenomic Mutation Rate

**DOI:** 10.1534/g3.120.401335

**Published:** 2020-06-11

**Authors:** Helmut Simon, Gavin Huttley

**Affiliations:** Research School of Biology, the Australian National University

**Keywords:** variance in mutation rate, context dependent mutation, ARMA models

## Abstract

We report work to quantify the impact on the probability of human genome polymorphism both of recombination and of sequence context at different scales. We use population-based analyses of data on human genetic variants obtained from the public Ensembl database. For recombination, we calculate the variance due to recombination and the probability that a recombination event causes a mutation. We employ novel statistical procedures to take account of the spatial auto-correlation of recombination and mutation rates along the genome. Our results support the view that genomic diversity in recombination hotspots arises largely from a direct effect of recombination on mutation rather than predominantly from the effect of selective sweeps. We also use the statistic of variance due to context to compare the effect on the probability of polymorphism of contexts of various sizes. We find that when the 12 point mutations are considered separately, variance due to context increases significantly as we move from 3-mer to 5-mer and from 5-mer to 7-mer contexts. However, when all mutations are considered in aggregate, these differences are outweighed by the effect of interaction between the central base and its immediate neighbors. This interaction is itself dominated by the transition mutations, including, but not limited to, the CpG effect. We also demonstrate strand-asymmetry of contextual influence in intronic regions, which is hypothesized to be a result of transcription coupled DNA repair. We consider the extent to which the measures we have used can be used to meaningfully compare the relative magnitudes of the impact of recombination and context on mutation.

Germline mutations are estimated to occur in humans with an average probability of $1.28\times 10^{-8}$ per site per generation, with ∼93% of these being point mutations (Roach *et al.* 2010; Jónsson *et al.* 2017a). Germline point mutations result in the creation of single nucleotide variants (SNVs) in a population. Evidence of genomic heterogeneity in mutation has been predominantly derived from between or within species analysis of genetic variation. For instance, mutation heterogeneity is implicitly supported by genomic heterogeneity in substitution rates (Hodgkinson *et al.* 2009; Ying *et al.* 2010) and in the relative abundance of nucleotides (Cuny *et al.* 1981). More recently, explicit *de novo* mutation studies (*e.g.*, Michaelson *et al.* 2012; Francioli *et al.* 2015; Smith *et al.* 2018) have been reported, and these too support a heterogeneity in mutation processes. The mechanistic origins of this mutation heterogeneity remain unclear. Likely candidates include a direct mutagenic influence of meiotic recombination and the effect of sequence neighborhood. Analyses of these potential contributors have predominantly drawn on SNV analyses and have led to inconsistent conclusions. Here we focus on development and application of a consistent analytical framework to quantify the relative importance of these different factors.

It has been established that the rate of mutation is non-uniform along the genome of humans and other species. The phenomenon of mutation heterogeneity was first observed in the bacteriophage T4 prior to the availability of DNA sequencing (Benzer 1961). Subsequent DNA sequence analyses of homologous genes revealed that G and C nucleotides were far more mutable than A and T nucleotides (Coulondre *et al.* 1978; Gojobori *et al.* 1982) and that mutation rates at these sites are influenced by neighboring bases (Bulmer 1986). We now have evidence that such non-uniformity can occur at scales ranging from individual nucleotides to multi-megabase sized regions (Hodgkinson and Eyre-Walker 2011). For instance, the heterogeneity of DNA composition suggests the existence of mutation rate heterogeneity at megabase scales and this has been supported by *de novo* mutation studies (Smith *et al.* 2018).

Previous studies have identified a number of key determinants of mutation rate, prominent among which are recombination rate and sequence neighbors. However, these studies have differed in certain of their conclusions. For example, relatively recent studies of *de novo* mutations have provided strong evidence of a direct causative effect of recombination on mutation (Arbeithuber *et al.* 2015). That nucleotide diversity is higher in regions of high recombination has been known for some time (Lercher and Hurst 2002; Duret and Arndt 2008). Whether this reflects a direct effect of recombination on mutation or an influence of selective sweeps in reducing diversity in regions of lower recombination is disputed (*e.g.*, Jensen *et al.* 2019). Previous population analyses used linear regression models (Lercher and Hurst 2002; Duret and Arndt 2008; Mugal and Ellegren 2011) to measure an association between mutation and recombination rates. Estimates from these approaches are potentially problematic as the methods used do not control for spatial auto-correlation of recombination and mutation rates across the genome.

The hypermutability of CpG dinucleotides (and the preponderance of genetic variation within this context) exemplifies the important influence of sequence context on the rate of mutation. In mammals and some other species, the transition mutation C→T, where the C is part of a CpG dinucleotide, is several times more common than mutations at other sites (Ehrlich and Wang 1981). The biochemical cause is known to be the spontaneous deamination of the highly unstable 5-methylcytosine (Coulondre *et al.* 1978). In mammals, methylation of cytosines is highly context dependent, occurring almost exclusively at CpG dinucleotides (Ramsahoye *et al.* 2000).

It has been demonstrated that all point mutations are affected to a greater or lesser effect by sequence context (Zhu *et al.* 2017). Using a log-linear model, Zhu *et al.* (2017) dissected the influence of nucleotide distance and the joint *vs.* independent influence of multiple nucleotides. These authors argued that the dominant neighborhood influences lay within ±2 for transition mutations, ±3 for transversions. Carlson *et al.* (2018) also found widespread influence of context on mutation types while restricting their analysis to extremely rare variants. Zhu *et al.* (2017) and Carlson *et al.* (2018) did not, however, directly address mutation rate or variance in the sense described above. An analysis using the $R^{2}$ metric of a linear model to measure the contribution of different contexts to variance (Aggarwala and Voight 2016) argued that nucleotides up to 3 sites distal can have a major influence on mutation rates. The linear regression model used by Aggarwala and Voight does not yield the maximum likelihood estimates of model parameters for this data, due to the binomial nature of the sampled data and the condition of heteroscedasticity consequently not being satisfied (Agresti, 2002, p. 120). Previous approaches also did not address issues of bias arising from neighborhood size. Bias will tend to inflate estimates of variance as a given data set of mutation counts is further subdivided into “buckets”, the number of which increases with neighborhood size *k* at the rate $4^{k}$.

One approach to quantifying the relative contributions of different factors on mutation is to measure the proportion of variance in mutation rate explained by them. Conversely, this measurement also indicates how much variance remains unexplained. Inherent in discussion of the variability of mutation rate is the assumption that each site in the genome has a specific mutation rate. Hence, we define the “total” variance in mutation rate as the conventional statistical variance of these quantities. This variance has been estimated by comparing variable positions in orthologous alignments of closely related species such as humans and chimpanzees (Hodgkinson *et al.* 2009). The probability of an SNV at a site is assumed to be some multiple *r* of the site mutation rate, with *r* fixed in each population. (The underlying mutation rates are assumed to be the same in humans and chimpanzees.) The variance in mutation rate can then be calculated from the number of SNVs that are observed at orthologous sites in both sequences. The conclusion from this approach was that there was substantial variance in the human mutation rate (∼64% of total variance) that was not explained by the interaction of a base with its immediately adjacent nucleotides (Hodgkinson *et al.* 2009). These authors minimized the potential role of larger sequence contexts, a conclusion that was later challenged by the results of other studies (Aggarwala and Voight 2016; Zhu *et al.* 2017).

Here we report work quantifying the contribution to the probability of human genome polymorphism that can be attributed to recombination and to sequence context at different scales. We use a Bayesian approach to quantify the uncertainty in our estimates of the variance and to overcome issues of bias which occur if a conventional estimator were used instead. Our results produce estimates of recombination induced mutation that are consistent with those from *de novo* mutation studies. We further establish that when considered across all point mutations, the influence of sequence neighborhood is dominated by 5-mer effects reflecting the markedly greater relative abundance of transition mutations. Finally, we emphasize the complexity in comparing the contributions to mutation of a state (sequence context) *vs.* the contribution to mutation of an event (recombination). Overall, we establish that a substantial proportion of mutation heterogeneity remains unaccounted for.

## Materials and methods

### Data

Data on human variants was sampled from Ensembl release 89 (for influence of context) and release 92 (for influence of recombination) variation databases (Cunningham *et al.* 2015) using the query capabilities of ensembldb3 (Huttley and Ying 2009). Variants were restricted to those identified by the 1000 Genomes (1KG) Project (1000 Genomes Project Consortium *et al.* 2015), but without restriction by source population. We did not analyze sex chromosomes, as structural differences from autosomes in mutation rate, recombination rate and effective population size mean that results from autosomes and sex chromosomes cannot meaningfully be aggregated.

deCODE provides estimated recombination rates averaged over 10-kilobase (kb) blocks. The files female_noncarrier.rmap, male_noncarrier.rmap and sex-averaged_noncarrier.rmap were downloaded from https://www.decode.com/addendum/ (Kong *et al.* 2010). These correspond to female, male and sex-averaged standardized recombination rates respectively. The proportion of the human genome covered by the deCODE estimates is given at Kong *et al.* (2010, Supplementary Table 2). The hg18 genome coordinates were mapped to GRCh38 using pyliftover, a Python implementation of UCSC LiftOver (Tretyakov 2013).

### Variance in probability of polymorphism due to recombination

In order to estimate variance in the probability of polymorphism that can be explained by recombination or by sequence neighborhood, we employ the SNV density as a surrogate. Counts of SNVs within the 10-kb blocks defined by deCODE were determined from the Ensembl variation database records. We excluded blocks where no SNVs were reported in Ensembl; blocks that were identified by deCODE as overlapping unsequenced regions; and blocks adjacent to these. The portions excluded in this way did not exceed 5% of any chromosome.

The relationship between recombination rate and SNV density may be confounded by spatial auto-correlation of these quantities along the genome. The impact of auto-correlation on the residuals of a linear model was confirmed by plotting the covariances of the residuals for blocks separated by up to 50 blocks using statsmodels (Seabold and Perktold 2010) (Figure S1). Allowing for auto-correlation in our model requires maintaining the lags between the 10-kb blocks and thus it was necessary to adjust regions with missing data. This was done using the Last Observation Carried Forward (LOCF) method (Molenberghs *et al.* 2014, p. 38). That is, for successive blocks excluded by our missing data criteria, SNV and recombination data from the immediate 5′ neighbor block were repeated.

Selection of the appropriate time-series model for the residuals depends on whether their distribution is stationary. The statsmodels (Seabold and Perktold 2010) implementation of the augmented Dickey-Fuller test (Mills, 2008, p. 79) was used to demonstrate stationarity of the residuals. (See also Figure S2.) Stationarity allows us to apply Wold’s decomposition theorem (Mills, 2008, p. 12) to conclude that the residuals can be approximated by an auto-regressive moving average (ARMA) model of some order (p,q) where *p* and *q* are non-negative integers and p>0. Optimal values of *p* and *q* were chosen by evaluating models for p≤10 and q≤4 using the statsmodels (Seabold and Perktold 2010) ARMA implementation to find which had the lowest value of the Akaike Information Criterion (AIC). (In the case of chromosome 9, the model with the second lowest AIC was used, as the lowest model confounded the subsequent Markov Chain Monte Carlo step.)

A Bayesian Markov Chain Monte Carlo (MCMC) approach implemented in the software package PyMC3 (Salvatier *et al.* 2016) was used to simultaneously estimate the slope, intercept and p+q ARMA parameters. This was developed to provide a more robust approach than iterative adjustment of the parameters (Mizon 1995) as is undertaken with, for example, the Cochrane-Orcutt procedure. The intercept *α* obtained from this process represents the model’s prediction of SNV density for genomic segments with a recombination rate of zero. Therefore, given the average SNV density $\bar{m}$, we can estimate the proportion of SNVs caused by recombination as $\hat{\rho }=\frac{\bar{m}-\alpha }{\bar{m}}$. Under a neutral model, for a realistically small mutation rate, the probability of an SNV at a site is some fixed multiple of the mutation rate at the site over the whole genome (Hodgkinson *et al.* 2009). Therefore $\hat{\rho }$ is also the proportion of mutations caused by recombination. Then if *x* is the number of mutations that occur in 1 Mb of DNA sequence in a specific generation and *y* is the number of recombination events occurring in that sequence in the same generation, $\hat{\rho }x$ is the expected number of new mutations in that segment caused by recombination. Since they must be caused by recombination events occurring in that generation, the expected number of mutation events per recombination event is $\hat{\rho }\frac{x}{y}$. Therefore multiplying $\hat{\rho }$ by the ratio of mutations per generation to recombination events per generation gives the average number of mutations produced by each recombination event. The estimated variance in SNV density due to recombination (${\hat{\sigma }}_{rec}^{2}$) is calculated as the difference between the total variance in SNV density and the sum of squares of the residuals. The ratio of this quantity to total variance in SNV rate is the proportion of variance in SNV rate attributable to recombination ($R^{2}$).

Our model does not take account of error in the estimation of recombination rates in the blocks. To determine the impact of this, we tried adding a normal perturbation of the recombination rates to the model. This made little difference to the posterior distribution, which we hypothesize is due to averaging the recombination rates over a large number of blocks.

### The variance in SNV density conditioned on context

We estimated the probability of polymorphism for all point mutation directions from all sequence contexts of size *k* that contained a central point mutation. The mean and variance can be obtained from these in a straightforward manner. The variance conditioned on different central bases or different point mutation directions can be measured by filtering the appropriate subset of the data. We now expand on our model and approach to estimation of the variance.

We denote by ${\mathbb{C}}_{k}(\cdot a\cdot )$ the set of $4^{k-1}$ sequence contexts with central base a∈{A,C,G,T}. The union, ${\mathbb{C}}_{k}$, of the four such sets contains the $4^{k}$ distinct *k*-mer sequences. As we are concerned with neighborhoods centered on a mutating base, *k* is an odd numbered integer with values of 3, 5, 7 or above.

For a sequence *S*, our model assigns to each site a fixed probability *m* of being polymorphic for an SNV and assumes that the mutation events for different sites occur independently (see Assumptions below). It is the variability of *m* that can be explained by context that is the object of the analysis.

For a context *c*, let $p_{c}$ be the proportion of sites in *S* matching it. We denote by $m_{c}$ the probability that a randomly selected site matching the context *c* will have a SNV at the central base. Then $m_{c}$ is the average SNV probability over the sites with context *c*. We denote by $\bar{m}$ the average SNV probability over the entire sequence. For any *k* we have:

$$\bar{m}=\underset{c\in {\mathbb{C}}_{k}}{\sum }p_{c}m_{c}$$

Then the total variance in SNV density accounted for by sequence neighborhoods of size *k* is:

$$\sigma_{k}^{2}=\underset{c\in {\mathbb{C}}_{k}}{\sum }p_{c}{\left(m_{c}-\bar{m}\right)}^{2}$$

This total variance can be partitioned into components consisting of variance attributable to each point mutation a→b as

$$\sigma_{k}^{2}(a\to b)=\underset{c\in {\mathbb{C}}_{k}(\cdot a\cdot )}{\sum }p_{c\left(a\right)}{\left(m_{c,a\to b}-{\bar{m}}_{a}\right)}^{2}$$ (1)

where $p_{c\left(a\right)}$ is the proportion of sites with base *a* whose context matches *c*; $m_{c,a\to b}$ is the probability of polymorphism arising from mutation of base *a* to base *b* in context *c*; and ${\bar{m}}_{a}$ is the probability that a site with base *a* will have an SNV.

We consider the proportion of contexts $p_{c}$ (and $p_{c\left(a\right)}$) as a fixed or known quantity, as contexts can be counted exactly with reasonable efficiency. We then estimate the values $m_{c}$ by ${\hat{m}}_{c}$, the empirical SNV density in context *c* (*cf*. Aikens *et al.* 2019). The estimated value of $\sigma_{k}^{2}$ is then given by:

$${\hat{\sigma }}_{k}^{2}=\underset{c\in {\mathbb{C}}_{k}}{\sum }p_{c}{\left({\hat{m}}_{c}-\hat{m}\right)}^{2}$$

where $\hat{m}$ is the empirical SNV density for the entire sequence. A similar equation applies if we condition on a specific point mutation direction a→b. For instance, we can further condition on C sites with 5′ G and G sites with 3′ C in order to isolate the CpG effect.

#### Assumptions:

Some of the assumptions made in the above model may be invalid in practice. We deal with this by filtering these conflicting cases from the data, as follows.

We have assumed that each site has a fixed probability of being polymorphic and that the resultant Bernoulli distributions are independent between sites. These assumptions fail if a site mutates more than once, since we allow the nucleotide to influence mutation rate. It similarly fails if a neighboring site mutates, since we allow context to influence mutation. We therefore only include those SNVs in our data set which are biallelic, with one allele being the ancestral allele; and for which there are no variants in the immediate neighborhood (4 bp on either side). (It is recognized that this does not eliminate the case in which subsequent mutations have occurred within the context and achieved fixation).

#### Bayesian model for estimation of variance due to context:

We use Bayesian conjugate priors to derive a posterior distribution for each instance of mutation direction within a particular context (*e.g.*, ACT→ATT mutation in the case of 3-mers). For each such case we have a count of *k*-mers (number of “trials”) and a count of variants at the central base of the *k*-mer (number of “successes”). The probability of polymorphism is given by estimating the probability parameter of a binomial distribution on these quantities. The conjugate prior for the binomial distribution is the beta distribution, so we use a Beta(1,1) distribution as a prior. We thus derive a posterior beta distribution for the mutation rate for the cell. We generate samples of the posterior distribution for the variance due to context by generating samples for the probability of polymorphism for each cell from the beta distributions and applying the right hand side of equation (1) to the samples to generate samples for the weighted variance.

This method requires that the number of mutation type and context pairs having no variants in the data are small. For such cells the posterior distribution on mutation rate would be Beta(1,1), the uniform distribution on [0, 1] and hence the variance in mutation rates would be inflated.

### Data availability

The authors state that all data necessary for confirming the conclusions presented in the article are represented fully within the article. Supplementary figures and tables are available at Zenodo https://zenodo.org/record/3875814. The preprocessed data used in this study are available at Zenodo https://zenodo.org/record/3874290 under the Creative Commons Attribution-Share Alike license. Larger data files are typically gzip compressed. Scripts and Jupyter notebooks developed specifically to perform the data sampling and analyses reported in this work were written in Python version _≥_3.5 and are freely available under the GPL at https://github.com/helmutsimon/ProbPolymorphism and at https://zenodo.org/record/3875855.

## Results

We estimated the contributions of recombination and context to the variance in SNV density using data from the Ensembl variation database (Cunningham *et al.* 2015). The SNV density for a sequence is defined as the number of qualified SNVs in the sequence divided by the sequence length. Only 1KG Project (1000 Genomes Project Consortium *et al.* 2015) variants were considered in the interests of consistency in SNV discovery. The point mutation direction from which a SNV was derived was inferred using the ancestral nucleotide state as recorded in Ensembl. Counts of filtered variants used by chromosome are shown at Supplementary Table S5.

### Effect of recombination on SNV density

We evaluated the relationship between recombination and SNV density using linear regression. Our aim was to recover the slope and intercept parameters from which other quantities of interest can be inferred. The slope parameter gives us the increase in SNV density for a given increase in recombination rate. In particular, a positive slope parameter indicates a positive effect of recombination on SNV density and hence mutation. The intercept parameter is the value of SNV density corresponding to a recombination rate of zero under the model. The estimated variance in SNV density due to recombination, which we denote by ${\hat{\sigma }}_{rec}^{2}$, is calculated as the difference between the total variance in SNV density and the sum of squares of the residuals. The ratio of this quantity to total variance in SNV rate is the proportion of variance in SNV rate attributable to recombination. This ratio is the standard metric $R^{2}$ (coefficient of determination), which measures the fit of a linear model in terms of explained variance in the observed data.

In modeling the influence of recombination, we used a partitioning of the genome into 10-kb segments for which average sex-averaged recombination rates were available (Kong *et al.* 2010). These rates are normalized relative to the average genetic distance over all of the 10-kb bins of 0.0116 centimorgans. SNV densities were derived from the number of SNVs in a segment.

We began by fitting an ordinary least squares linear regression (OLSLR) model to the data. Use of an OLSLR model for inference requires residuals to be mutually independent, in particular that there is no correlation between adjacent bins along the genome (spatial auto-correlation). By analyzing the residuals from an OLSLR model we identified a high level of auto-correlation (see Supplementary Figure S1) and determined that they were most appropriately modeled by an ARMA(p,q) model, where *p* and *q* are non-negative integers and p>0 (see Materials and methods). For each chromosome, we tested a range of ARMA models to find the one with the lowest Akaike Information Criterion (AIC) score for the data. The slope, intercept and ARMA error parameters were simultaneously estimated using a Bayesian Markov Chain Monte Carlo (MCMC) approach (see Materials and methods), obviating the need for iterative “adjustment” steps.

**Figure 1:**
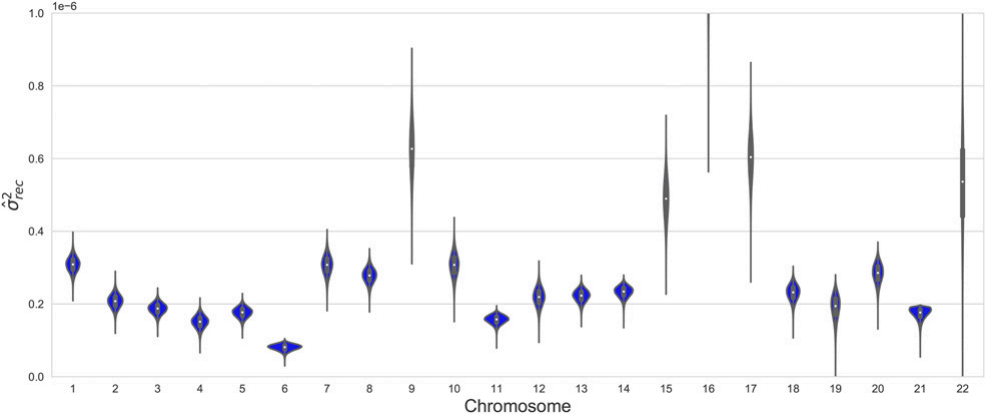
Estimated variance in SNV density attributable to recombination by chromosome. The variances ($\sigma_{rec}^{2}$) are estimated by fitting a linear model to each chromosome, with residuals modeled by an ARMA(p,q) model optimized for each chromosome. The variance due to recombination is the difference between total variance and the variance not explained by the model.

The above process was applied to all chromosomes individually. The estimates for variance in SNV rate due to recombination (${\hat{\sigma }}_{rec}^{2}$) are shown as violin plots in Figure 1. It can be seen that there were some significant differences in the variance estimates for different chromosomes. In particular, chromosomes 9, 15, 16, 17 and 22 show significantly higher levels of variance in SNV density due to recombination. There were also significant differences in estimates of the slope and intercept parameters (see Supplementary Table S1). These differences between chromosomes precluded estimation of the influence of recombination across the genome as a whole. Specifically, using a model which set the slope and intercept parameters to be common across chromosomes while allowing differing ARMA models and parameters for each chromosome resulted in a y-intercept that was larger than the average SNV density, which is inconsistent with results from individual chromosomes. Modifications of this approach that used the sex-specific recombination maps did not result in any substantial differences (results not shown).

Estimates for the slope (change in SNV density per centimorgan) provided strong evidence for a positive effect of recombination on SNV density across all chromosomes. The estimates ranged from 0.0061 for chromosome 4 to 0.0092 for chromosome 14 (see Supplementary Table S1). The corresponding 95% credibility intervals (hereafter CI) of these estimates were 0.0044-0.0078 and 0.0057-0.010 respectively. For all chromosomes tested, the posterior probabilities that the slope was ≤0 were $\leq 10^{-3}$ (estimated from the MCMC variates).

In the linear model, the y-intercept represents the predicted SNV density for a recombination rate of zero. Estimates for the y-intercept ranged from 0.0251 (95% CI of 0.0246-0.0255) for chromosome 1 to 0.0285 (95% CI of 0.0257-0.0313) for chromosome 16. The difference between the mean SNV density and the y-intercept parameter is more significant as it represents the difference between the average observed SNV density calculated and the observed data and the SNV density predicted for a recombination rate of 0. That is, this difference measures the part of the SNV density that can be attributed to recombination under the model. Dividing the difference by mean SNV density gives the proportion of SNVs that can be attributed to recombination ($\hat{\rho }$, Materials and methods). This quantity varied between 0.24% for chromosome 4 and 0.59% for chromosome 8.

We also examined the extent to which the effect of recombination on SNV density differed for the 12 point mutations directions for each chromosome. As an example, results for chromosome 1 are shown in Table 1. We accepted that recombination has had a positive effect on mutation when the posterior probability that the slope was less than zero was found to be less than 0.05. On this basis, the mutations for which recombination influenced mutation in Chromosome 1 comprise all four transitions (C→T, T→C, A→G, G→A) and the N→S transversions C→G, G→C, T→G and A→C.

**Table 1:** Analysis of the linear relationship between recombination rates and SNV densities for chromosome 1 disaggregated by mutation direction. ‘SNV Density’ is the SNV density for that mutation direction (conditioned on ancestral allele); ‘Probability’ is the posterior probability that the slope parameter from the linear regression is less than zero; ‘${\hat{\sigma }}_{rec}^{2}$’ is the estimated variance due to recombination and ‘Lower CL 95%’ and ‘Upper CL 95%’ are the limits of the 95% credibility interval for ${\hat{\sigma }}_{rec}^{2}$

Mutation	SNV Density	Probability	${\hat{\sigma }}_{rec}^{2}$	Lower CL 95%	Upper CL 95%
C→T	0.0247	0.0000	3.7e-07	2.9e-07	4.4e-07
G→A	0.0246	0.0000	3.8e-07	3.0e-07	4.4e-07
T→C	0.0124	0.0000	5.0e-08	4.4e-08	5.3e-08
A→G	0.0125	0.0000	8.2e-08	7.1e-08	9.1e-08
C→G	0.0047	0.0004	2.6e-09	1.4e-09	3.1e-09
G→C	0.0047	0.0022	1.0e-09	4.4e-10	1.2e-09
T→G	0.0030	0.0109	9.3e-10	1.9e-10	1.3e-09
A→C	0.0030	0.0168	9.4e-10	9.2e-11	1.4e-09
T→A	0.0029	0.9472	−2.8e-10	−9.5e-10	1.9e-12
A→T	0.0029	0.3697	9.6e-12	−5.6e-10	2.3e-10
C→A	0.0054	0.9140	−5.3e-10	−2.0e-09	−1.3e-12
G→T	0.0054	0.9290	−8.9e-10	−2.8e-09	6.2e-11

Since the estimated variance in SNV density due to recombination is calculated as the difference between the total variance in SNV density and the sum of squares of the residuals, it will be negative if the model fit is worse than for a line with zero slope. This is likely to occur when the ‘Probability’ value is significantly greater than zero and we reject the model.

**Figure 2:**
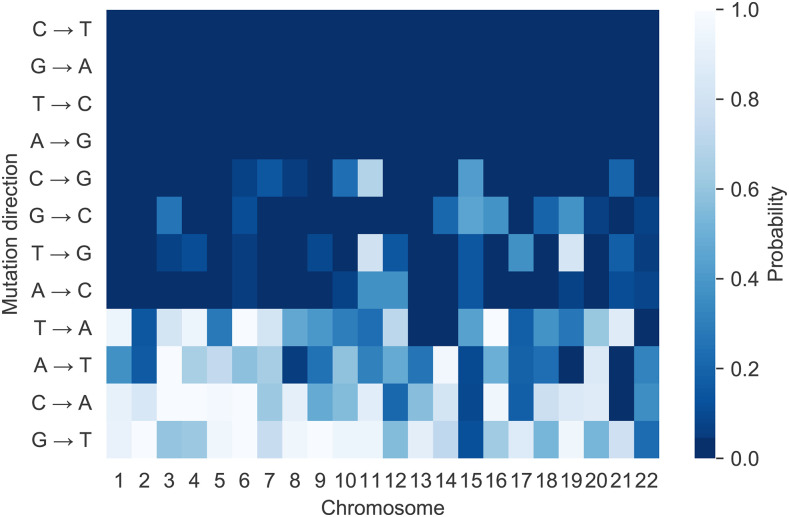
Evidence for the effect of recombination on mutation by mutation direction and chromosome. ‘Probability’ is the posterior probability that the slope parameter from the linear regression is less than zero. A darker shade indicates a high probability that SNV density has a positive linear relationship with recombination. Cells meeting the 0.05 threshold are in the deepest blue.

For SNVs derived from transition mutations, evidence for an association with recombination rate was consistent across all chromosomes (Figure 2 and Supplementary Table S2). This was not the case for the transversion mutations. For SNVs derived from transversions, evidence of an influence of recombination ranged from inconsistent to none. For instance, for transversions to G/C, the posterior probabilities for most chromosomes met our 0.05 threshold. In contrast, there was no evidence of an influence of recombination on transversions to A/T for most chromosomes. Additionally, if a mutation type appears to be influenced by recombination, so does its strand-symmetric counterpart. However, the values for variance due to recombination for a mutation and its strand-symmetric counterpart, while of similar magnitude, do not necessarily coincide, even using the 95% CI. For all chromosomes the mutations with the highest variance due to recombination are C→T and G→A, the same as are subject to the CpG effect.

### Variance in SNV density due to context

In our analysis of variance in SNV density due to context, we restricted ourselves to intronic 1KG Ensembl variants, to reduce confounding due to selection. All SNVs and contexts were oriented with respect to the annotated strand of the gene. For consistency with our assumption that each site has a fixed mutation rate, only biallelic variants separated by ≥4 nucleotides from another SNV were considered. Rather than use a conventional sample or plug-in estimator of the average SNV density for each context, we worked with samples from a posterior (beta) distribution to the binomial likelihood function. This allowed us to sample and graph posterior distributions for variance due to context, showing the uncertainty in the parameter estimates (see Materials and methods). It also allowed us to calculate the (posterior) probabilities of particular conditions by counting the proportion of MCMC variates satisfying the condition.

To evaluate the relationship between sequence context and the probability of a SNV requires further definition of SNV density. For a specific sequence context of size *k* (including the middle position), there are $4^{k}$ distinct contexts (hereafter *k*-mers). To illustrate calculation of SNV density, consider the 3-mer ACA. We estimated the SNV density for ACA as the number of occurrences of ACA for which the middle position had an SNV divided by the total number of occurrences of the *k*-mer ACA. This can be further partitioned into the different point mutations from C. The estimated variance attributable to sequence context of size *k*, which we denote by ${\hat{\sigma }}_{k}^{2}$, is thus the variance computed across all $4^{k}$ such densities (see Materials and methods).

**Figure 3:**
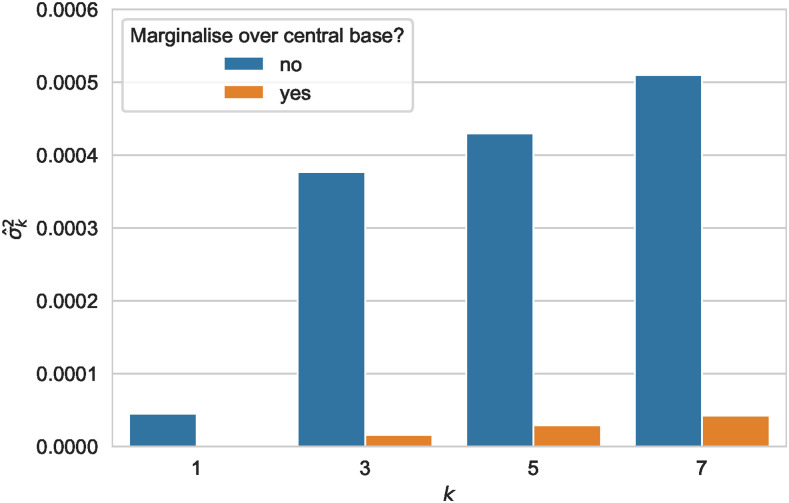
The interaction between a mutating base and its neighborhood dominates variance in SNV density. The component of ${\hat{\sigma }}_{k}^{2}$ attributable to neighborhood alone (*i.e.*, marginalised over the central base) is shown in tan. This contrasts with the full value of ${\hat{\sigma }}_{k}^{2}$ (shown in blue), which includes the interaction between a mutating base and its neighborhood.

The values of ${\hat{\sigma }}_{k}^{2}$ for k= 1, 3, 5 and 7 are shown by the blue bars in Figure 3 for 1-mers to 7-mers. The case of k=1 shows variance conditioned solely on ancestral base. We necessarily observe an increase in variance with increasing *k*, but the increments diminish markedly after 3. It is noteworthy that the variance due to the central base alone only comprises ∼12% of the variance due to 3-mers. The total variance due to 7-mers is ∼35% greater than that due to 3-mers. Variance in SNV density calculated in this way includes the influence of the central mutating base itself and the interaction between that central mutating base and its neighborhood. To investigate the relative influence of these elements further, we show, using the tan bars, the values of ${\hat{\sigma }}_{k}^{2}$ marginalised over the central base (Figure 3). That is, we evaluated the influence of the flanking nucleotides alone, by pooling counts of variants and *k*-mers which share the same central base and calculating the variance of the SNV frequencies for the bins formed in this way. We see that while these values are much lower than the unmarginalized values, the relative magnitude of the increments from 3-mer to 5-mer and from 5-mer to 7-mer are larger. We can conclude that the greater part of the unmarginalized variance due to 3-mers is explained not by the independent actions either of the central base or of the flanking bases, but by the interaction of the central base with its immediately adjacent neighbors. Furthermore, this interaction between a mutating base and its immediate neighbors is the largest contribution to variance for all values of *k* considered. As would be expected, a large component of this is due to the CpG effect (including its strand-symmetric counterpart) which we estimated as 0.00028, ∼ 54% of the variance due to 7-mers.

**Figure 4:**
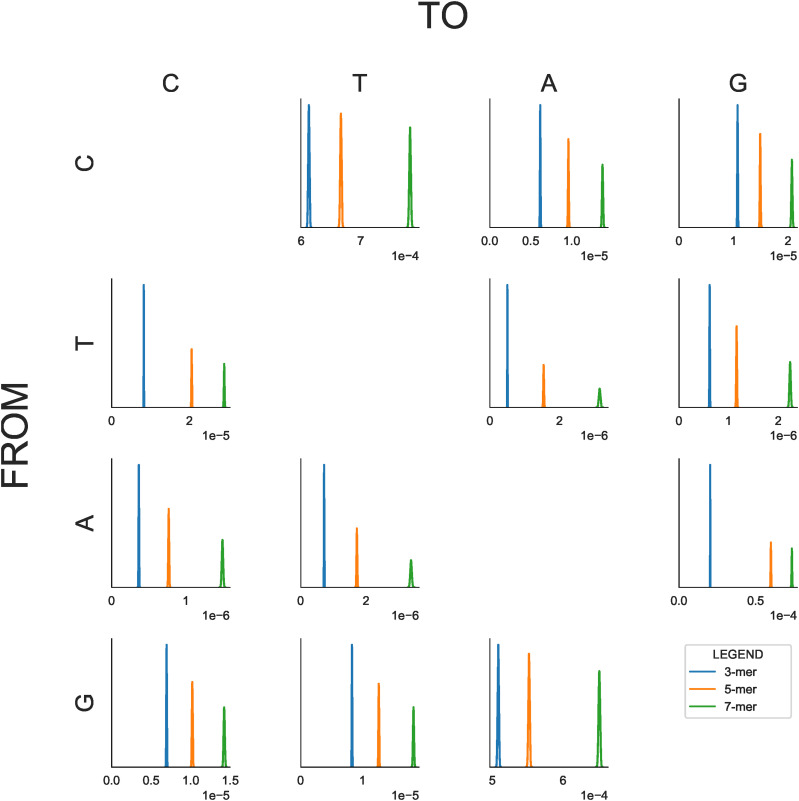
Posterior distributions of the variance of SNV density (x-axis) conditioned on 3-mer, 5-mer and 7-mer contexts for each of 12 mutation profiles. The Row/Column labels correspond to the *from* and *to* nucleotides. Note that the x-axes (${\hat{\sigma }}_{k}^{2}(a\to b)$, estimated variance due to context) and y-axes (probability density) scales vary between plots. In particular the x-axes for C→T and G→A mutations do not include the origin.

We also analyzed the variance due to context for each of the point mutations separately. The results are shown in Figure 4 as posterior distributions for the variance due to context. (Values for the posterior mean are shown at Supplementary Table S3.) The increment in variance from 5-mer to 7-mer is greater than or approximately equal to that from 3-mer to 5-mer for all point mutations, with the exceptions of T→C / A→G transitions. In the case of transversions, the variance due to 7-mers is approximately two to three times that due to 3-mers. The strong relative influence of 7-mers and 5-mers for transversions may appear to be at odds with the results aggregated over point mutations (Figure 3). However, since Figure 4 considers each point mutation separately, the central or ‘from’ base is fixed and the interaction between the central base and its immediate neighbors does not make a contribution. Thus the impact of increasing *k* is more similar to that of the marginalised quantities (Figure 3).

**Figure 5:**
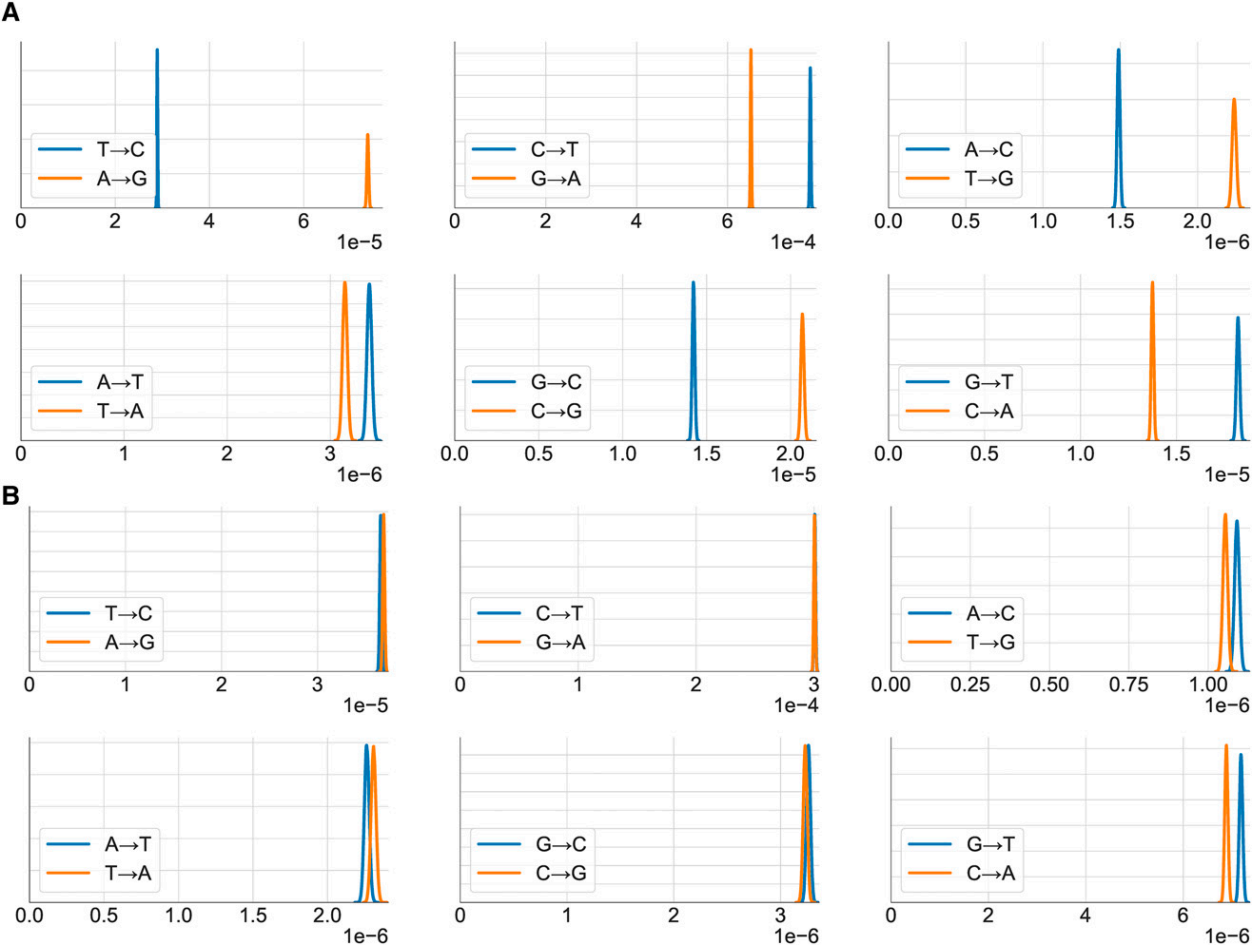
Variance in SNV density for specific mutation directions is strand-asymmetric for intronic regions, but strand-symmetric for intergenic regions. We show posterior distributions of ${\hat{\sigma }}_{7}^{2}$ for each of the 12 mutation directions (x-axis), with strand-symmetric pairs shown on the same axes. The y-axes show probability density. (a) Intronic regions. (b) Intergenic regions.

Examination of Figure 4 suggests that contextual influence does not always operate in a strand-symmetric manner. We investigated this further by plotting intronic mutations together with their strand-complements for the 7-mer case (Figure 5a). This demonstrates evidence of strand-asymmetry for all mutation types. This was especially marked for T→C / A→G transitions. Our criterion for rejecting strand-symmetry was that the 97.5 percentile of one of a pair of strand-complementary mutations was less than the 2.5 percentile of the other. As a control, we performed a similar analysis for intergenic regions. The results (Figure 5b) are generally consistent with the operation of strand-symmetric processes in intergenic regions. The pair of mutations G→T and C→A appear to be strand-asymmetric by our criterion and may be an exception or an artifact.

## Discussion

A multitude of processes contribute to spontaneous mutagenesis. One approach to establishing the relative importance of these is to use genomic heterogeneity in SNV density, which under a neutral model can be presumed to arise from the non-uniform action of factors contributing to mutation rate. Identifying an association between candidate factors and SNV density can provide evidence of their effect on mutation. In this paper we developed an approach of estimating the variance in SNV density conditioned on either recombination rate or on sequence context of various sizes. We confirmed an association between recombination and SNV density. Our analyses of contextual influences on SNV density demonstrated that the effect of context size differed between transition and transversion point mutations and did not always operate in a strand symmetric manner.

Our conclusions are derived using a model of mutation that is necessarily a simplification of a complex reality. We have assumed that each genomic site is associated with a specific mutation rate and that mutation events at different sites occur independently (*e.g.*, Hodgkinson *et al.* 2009). We stated earlier that achieving consistency with these assumptions requires some filtering of the data (see Assumptions in Materials and Methods). It is also known that mutations at different sites are not always independent due to the distinct phenomena of ectopic gene conversion (Harpak *et al.* 2017) and of “mutation clusters” (Michaelson *et al.* 2012). The assumption that each site has a fixed mutation rate is also a simplification as it is known that such mutation rates are influenced by external factors, notably parental age (*e.g.*, Francioli *et al.* 2015).

### The influence of recombination on mutation

We investigated the relationship between recombination and SNV density by testing for an association between the average recombination rate evident in 10-kb genomic segments and the SNV density in those segments. Due to substantial auto-correlation of mutation and recombination rates between neighboring segments (Figure S1), we derived estimates of the variance using a linear regression model that was modified to incorporate this auto-correlation. For all chromosomes examined, the posterior probabilities that the slope was ≤0 were $\leq 10^{-4}$. This provides strong evidence of a positive correlation.

Taking the step from inferences about SNV density to inferences about mutation rate generally relies on an assumption that the ratio of the mutation rate to the probability of polymorphism is constant over all sites in a given population of genomes (*cf*. Hodgkinson *et al.* 2009). On this basis, we can apply the ratio of the estimated point mutation rate in human chromosomes (Jónsson *et al.* 2017a) to the average SNV density obtained from our data to express our above estimates for the slope in terms of the per base pair mutation rate per centimorgan. These results range from $2.17\times 10^{-9}$ (chromosome 21) to $4.13\times 10^{-9}$ (chromosome 17) (Table S1). However, such a constant relationship between SNV density and mutation rate applies to a neutral model of evolution that does not take account of at least two known factors: selective sweeps and GC-biased gene conversion (gBGC). Both of these factors are themselves influenced by the recombination rate in a region. Thus a number of factors potentially contribute to the relationship between recombination and SNV density. We aim to address the question of the contribution of a direct mutagenic effect of crossovers relative to other factors including selective sweeps and gBGC. Our approach using linear models incorporating ARMA distributions assists in this by providing a more accurate quantification of the overall relationship between recombination rate and SNV density.

We hypothesize that the direct mutagenic effect of recombination makes a significant contribution relative to the other identified factors. We compare our results to those of a recent study of *de novo* mutations which estimated the probability of a recombination event (crossover) causing a mutation at $\hat{\rho }$=0.29%, with a 95% confidence interval of 0.17–0.47% (Arbeithuber *et al.* 2015). Such direct measurements by estimation of these rates from *de novo* mutation studies constitute in principle a “gold standard”, subject to experimental limits and small sample sizes. If *y* is the average point mutation rate of the DNA sequence and *x* is the average rate of crossovers, the proportion of the mutation rate that is directly caused by crossovers is $\frac{\rho x}{y}$. Setting $y=1.19\times 10^{-8}$ (Jónsson *et al.* 2017b) and $x=1.16\times 10^{-8}$ (Kong *et al.* 2010), we estimate the proportion of the mutation rate directly caused by crossovers at 0.28%. If we ignore the effect of selective sweeps and gBGC by retaining the simplifying assumption that SNV density is a fixed multiple of the mutation rate regardless of location, we would predict that 0.28% of SNVs are also directly caused by crossovers. Comparing this to the last column of Supplementary Table S1 we see that this estimate of 0.28% for the proportion of SNVs caused directly by crossovers accounts for over 50% of the proportion of additional SNVs associated with recombination rate as derived from our modified linear model. The latter quantity measures the influence of recombination by all mechanisms, thus this outcome provides empirical support for the hypothesized importance of the direct mutagenic effect of recombination.

Our estimates for the slope in terms of the base pair mutation rate per centimorgan are somewhat greater than the overall figure of $∼1.5\times 10^{-9}$ obtained in Hellmann (2005). We note that Hellmann (2005) addressed the issue of spatial auto-correlation by means of the Cochrane-Orcutt correction (Kutner *et al.* 2005), which is applicable to an auto-regressive (AR) model. AR models are nested within ARMA models as a special case. Our analysis of the residuals (data not shown) demonstrates that, taking the case of chromosome 1, the optimal ARMA model has a superior AIC (∼−184,554) to the optimal AR model (∼−184,493). On the other hand, our estimate of the probability that a single recombination event causes a mutation is very different from the estimate in Hellmann (2005), which was also based on a population analysis. In contrast to our approach, which addresses this relationship in terms of the intercept estimated from the linear model, Hellman’s estimate was based on the slope. Specifically, the slope of the linear regression was estimated at $∼1.5\times 10^{-9}$ mutations per base pair per centimorgan, that is, every 1% increase in the recombination rate in 1 Mb of sequence generated an additional ∼0.0015 mutations per Mb. The number of mutation events per recombination event is then taken as 0.0015/0.01=0.15, which is two orders of magnitude greater than the empirical estimates of Arbeithuber *et al.* (2015). This argument implicitly treats all recombination-induced mutations as having been caused by recombination events in the most recent generation. It therefore ignores recombination-induced mutations caused by recombination events in previous generations.

The use of an ARMA distribution to model the residuals, rather than fitting a conventional linear regression, made a major difference to the results. To illustrate this, we repeated the analysis using an OLSLR model (Supplementary Table S4). Comparing these estimates with those from the ARMA model (Supplementary Table S1) shows marked differences in all parameter values. In particular, the variance due to recombination (${\hat{\sigma }}_{rec}^{2}$) was some orders of magnitude larger in the OLSLR model. Critically, the intercept parameter was consistently lower for the OLSLR model and as a direct result the estimation of the number of mutations per recombination event was consistently greater for the OLSLR model by a factor of around 2. As correlations are given by the square root of $R^{2}$ in a linear model, the discrepancies we identify raise doubts about the accuracy of estimates of correlation between recombination rates and substitution rates in studies that do not compensate for spatial auto-correlation (Lercher and Hurst 2002; Duret and Arndt 2008; Mugal and Ellegren 2011).

Our results indicate the effect of recombination depended on mutation direction with some mutations exhibiting no association (*e.g.*, N→W transversions). This dependence on point mutation direction has been noted by other authors and our results are generally consistent with previous observations derived from substitution data (Duret and Arndt 2008). In particular, the mutation types seen to be most influenced by recombination were C→T and G→A, the same types that are subject to the CpG effect. Arbeithuber *et al.* (2015) reported all but one of the 17 *de novo* mutations found in molecules with a crossover were of one of these two types (as were the three mutations found in non-crossover controls). A possible explanation is that recombination magnifies the CpG effect by effecting a temporary local strand separation, since the deamination of 5-methylcytosine is over 60 times more rapid in single-stranded than in double-stranded DNA (Ehrlich *et al.* 1986; Zhang and Mathews 1994). The finding that the relationship between SNP density and recombination rate varied between mutation directions, including the absence of a correlation for some mutation directions, has no obvious explanation in terms of selective sweeps. This provides further support for the relative significance of the direct mutagenic effect of recombination.

**Figure 6:**
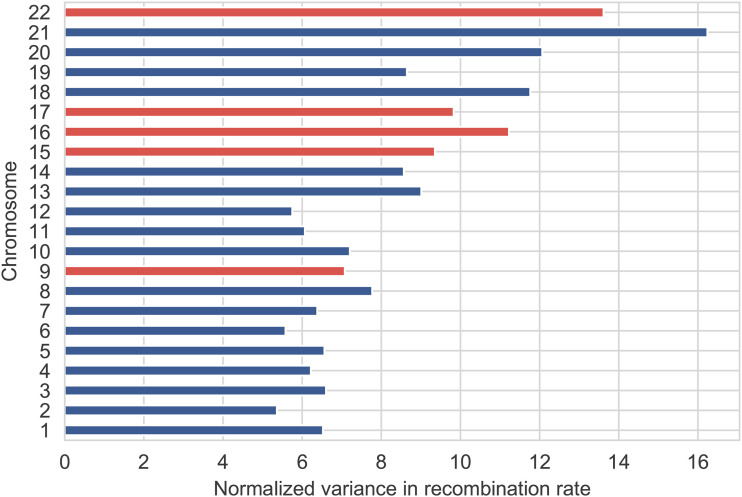
Variance in recombination rate by chromosome. The variance in recombination rate is calculated from average recombination rates for 10-kb bins, normalized by the mean recombination rate of the entire genome.Chromosomes with a higher value of ${\hat{\sigma }}_{rec}^{2}$ are shown in red.

Recombination will only contribute to variance in SNV density insofar as it occurs heterogeneously along the genome. Neither the proportion of mutation events occurring in a single generation that are caused by recombination nor the probability that a recombination event gives rise to a mutation event (both ∼0.004) are affected by the distribution of recombination rates along the genome. For this reason, these quantities arguably provide a better measure of the direct effect of recombination on mutation. A comparative analysis of ${\hat{\sigma }}_{rec}^{2}$ given in Figure 1 showed some difference between chromosomes, with chromosomes 9, 15, 16, 17 and 22 having a significantly higher value of ${\hat{\sigma }}_{rec}^{2}$ than the other chromosomes. One possible explanation would be that recombination rates are more heterogeneous along these chromosomes. One measure of heterogeneity is the variance of the recombination rates, which is shown in Figure 6. We see that chromosomes 15, 16, 17 and 22 do have relatively high variance, but not significantly higher than chromosome 13, while the variance for chromosomes 18, 20 and 21 is greater than for chromosomes 15, 16 and 17. Chromosome 9, on the other hand, does not have a relatively high variance in recombination rate. Thus while variance in the recombination rate may contribute to variance in ${\hat{\sigma }}_{rec}^{2}$, the explanation appears to be more complex. The extent of segmental duplication in chromosomes may also be a factor, as it correlates significantly with ${\hat{\sigma }}_{rec}^{2}$. The autosomes with the highest rate of segmental duplication are 7, 9, 15, 16, 17 and 22 (Zhang *et al.* 2004). As regions of segmental duplication are susceptible to ectopic gene conversion, this process may explain an increase in ${\hat{\sigma }}_{rec}^{2}$.

Another feature of Figure 1 is that chromosomes 9, 15, 16, 17 and 22 also have greater spread in their posterior distributions. This is likely to result directly from the fact that the variance due to recombination ($R^{2}$) is greater in these cases (Wishart *et al.* 1931; Olkin and Finn 1995).

### The influence of context on mutation

Our approach to analyzing the influence of context on mutation has two main features: using a rich database of human variants identified by the 1KG Project (1000 Genomes Project Consortium *et al.* 2015) and applying directly the concept of variance in mutation rate due to context as described in Materials and methods. This method is particularly suited to considering the issue of the effect on mutation of contexts of differing sizes.

A number of authors have variously dismissed (Krawczak *et al.* 1998; Hodgkinson *et al.* 2009) or made strong claims for (Aggarwala and Voight 2016; Zhu *et al.* 2017) the influence of contexts beyond 3-mers on mutation. Figure 3 shows that the increases from ${\hat{\sigma }}_{3}^{2}$ to ${\hat{\sigma }}_{5}^{2}$ to ${\hat{\sigma }}_{7}^{2}$ are relatively small. This is because the variance due to 3-mers incorporates the interaction between the central base of the 3-mer and its two flanking bases, which is the largest contributor to variance in SNV rates due to context. We found that ∼54% of ${\hat{\sigma }}_{7}^{2}$ was due to the CpG effect.

For analysis of an individual point mutation, the central base is fixed and thus there are no explicit interactions with neighboring bases. In this case, which we have referred to as fixed marginals (Figure 4), the additional variance added by 5-mers over 3-mers and, to an even greater extent, by 7-mers over 5-mers is substantial. These results may appear to support previous findings that the variance due to 7-mers is highly significant (Aggarwala and Voight 2016). However, the methods used in that work differ markedly to ours. When considering the influence of *k*-mers on the probability of observing an SNV, Aggarwala and Voight (2016) fitted a linear model to binomial data, which will not yield a valid maximum likelihood estimate of the slope and intercept parameters (Agresti 2002, p. 120). In calculating the relative influence of 3-mers and 7-mers, they used linear regression to predict the 7-mer SNV densities from the 3-mer SNV densities and calculated the $R^{2}$ metric on this regression. This metric is mathematically the same as the ratio of the following quantities: sum of squares difference between the 3-mer SNV densities and the overall mean SNV density; and, the sum of squares difference between the 7-mer SNV densities and the overall mean SNV density. This ratio is in turn the ratio of variance in 3-mer SNV densities to that of 7-mer SNV densities (without being weighted by frequency of context.) This appears to account for some similarity of their results to ours.

In contrast to Aggarwala and Voight (2016), Zhu *et al.* (2017) used a log-linear model for estimating the information content of neighboring bases. This approach is appropriate in modeling binomial data and also allowed comparison of the effect of different *k*-mers as measured by information content rather than traditional sum-of-squares variance. An advantage is that the joint effect of neighboring nucleotides can be distinguished from the independent effects of each. That work likewise identified neighboring nucleotides as distant as 4 bases away (hence k=9) as associated with some transversion point mutations (Zhu *et al.* 2017).

The strong influence of 7-mer contexts apparent when conditioning on the central mutating base raises the question of whether this is due to specific hypermutable 7-mer contexts. Our investigation failed to identify any such 7-mer contexts that were not attributable to CpG hypermutability. The most mutagenic context was NNACGNN. This sequence is of course subject to the CpG effect and the 5′-A has a positive association on C→T mutations independent of the 3′ base (see Zhu *et al.* 2017, Figure 2). The incidence of ACG trinucleotides was ∼7% of that expected from the individual nucleotide frequencies.

Figure 5a provides evidence of strand-asymmetry in the variance due to contextual influence for all 12 point mutation directions. It has been conjectured that such strand-asymmetry is caused by transcription coupled DNA repair (TCR) (*e.g.*, Hwang and Green 2004). TCR is a strand-asymmetric process which occurs in actively transcribed genes when an RNA polymerase (RNAP) translocating along a DNA strand encounters a distorting lesion or other local factor that retards its forward progress and may cause it to recruit nucleotide excision repair proteins (Spivak and Ganesan 2014). Sequence context is known to be involved in factors that can pause or arrest RNAPs (Spivak and Ganesan 2014). In their phylogenetic analysis of substitution rates, Hwang and Green (2004) found that T→C substitution rates were higher than those for A→G and C→T substitution rates were higher than those for G→A. Our analysis of SNV data differs from this in showing A→G to have a significantly higher SNV density than T→C while C→T SNVs had only marginally greater SNV density compared to G→A (Table S3). Figure 5a shows the same pattern in variance due to context: ${\hat{\sigma }}_{7}^{2}\left(C\to T\right)>{\hat{\sigma }}_{7}^{2}\left(G\to A\right)$ and ${\hat{\sigma }}_{7}^{2}\left(A\to G\right)>{\hat{\sigma }}_{7}^{2}\left(T\to C\right)$. Hwang and Green (2004) also showed transcription-associated mutational asymmetry to be influenced by context for transitions. Our results indicate that such influence occurs to some significant degree for all mutation directions. Overall, it appears that a substantial association exists between TCR and variance in SNV density.

## Conclusion

We have demonstrated that estimating the variance in SNV density due to context can discriminate the effect of contexts of different sizes. This was done from three perspectives: considering the 12 point mutation directions separately; aggregating over these directions while marginalizing over the central allele; and aggregating over these directions without marginalizing over the central allele (measured by ${\hat{\sigma }}_{k}^{2}$). The perspective adopted has a marked influence on estimates of relative influence. For example, results aggregated over mutation direction will be dominated by the more abundant transition mutations and in particular, by the CpG effect. Our approach has clarified the relationship between results from these different perspectives and, in particular, has demonstrated the dominant effect of the interaction between a central allele and its immediate neighbors. The use of Bayesian posterior distributions was able to give a high degree of certainty to conclusions about the strand-asymmetry of contextual influence in intronic regions. Further, our methods are driven solely by varying SNV densities between contexts and are not influenced by the distribution of *k*-mers within the genome.

We also quantified variance in SNV density due to recombination. However, a direct comparison of this quantity with the variance in SNV density due to context has some limitations. We measured variance in SNV density due to recombination at the scale of 10-kb DNA blocks. This does not take account of any variance due to recombination that exists within 10-kb blocks. This limitation is not easily overcome as there is presently no data for fine scale recombination at the individual base level.

We note that the quantitative impacts of recombination and context on mutation are conceptually difficult to compare meaningfully, as context is a state and recombination an event. For this reason, the proportion of mutation events caused by recombination and the probability that a recombination event gives rise to a mutation event (both _∼_0.004) are better measures of the direct impact of recombination on mutation. Our estimate that recombination only accounts for ∼0.4% of the average mutation rate makes recombination appear a relatively minor contributor to mutation rate overall. However, recombination is concentrated in hotspots, typically 1 - 2 kb in length, in which the recombination rate can commonly be 50 or more times higher than average (The International HapMap Consortium 2005). In such regions, recombination would account for ∼20% or more of the mutation rate.
